# Supplementary material for: ECMO in Myocardial Infarction-Associated Cardiogenic Shock: Blood Biomarkers as Predictors of Mortality
Source: Diagnostics (Basel). 2023 Dec 17;13(24):3683. doi: 10.3390/diagnostics13243683 (PMC10742636; doi:10.3390/diagnostics13243683)
Supplement: Supplementary file 1 [file diagnostics-13-03683-s001.zip › diagnostics-2773555-supplementary.pdf]

Article

ECMO in Myocardial Infarction-Associated Cardiogenic Shock: Blood Biomarkers as Predictors of Mortality

Supplementary material

Contents:

Table S1. STROBE Statement - Checklist of items that should be included in reports of cohort studies
Table S2. Identification of risk factors for death: univariate analysis (n = 188)
Table S3. Identification of risk factors for death: multivariate analysis (Procalcitonin on day three; n = 188)
Table S4. Identification of risk factors for death: multivariate analysis (Procalcitonin on day four; n = 188)
Table S5. Identification of risk factors for death: multivariate analysis (Procalcitonin on day five; n = 188)

Table S1. STROBE Statement - Checklist of items that should be included in reports of cohort studies

No.	Item	Recommendation	Page
	**Title and abstract**		
1		(*a*) Indicate the study's design with a commonly used term in the title or the abstract	1
		(*b*) Provide in the abstract an informative and balanced summary of what was done and what was found	1
	**Introduction**		
2	Background/rationale	Explain the scientific background and rationale for the investigation being reported	1-2
3	Objectives	State specific objectives, including any prespecified hypotheses	2
	**Methods**		
4	Study design	Present key elements of study design early in the paper	2-3
5	Setting	Describe the setting, locations, and relevant dates, including periods of recruitment, exposure, follow-up, and data collection	2-3
6		(*a*) Give the eligibility criteria, and the sources and methods of selection of participants. Describe methods of follow-up	2-3
	Participants	(*b*) For matched studies, give matching criteria and number of exposed and unexposed	2-3
7	Variables	Clearly define all outcomes, exposures, predictors, potential confounders, and effect modifiers. Give diagnostic criteria, if applicable	2-3
8*	Data sources/ measurement	For each variable of interest, give sources of data and details of methods of assessment (measurement). Describe comparability of assessment methods if there is more than one group	2-3
9	Bias	Describe any efforts to address potential sources of bias	2-3
10	Study size	Explain how the study size was arrived at	-
11	Quantitative variables	Explain how quantitative variables were handled in the analyses. If applicable, describe which groupings were chosen and why	-
12		(*a*) Describe all statistical methods, including those used to control for confounding	3
		(*b*) Describe any methods used to examine subgroups and interactions	3
		(*c*) Explain how missing data were addressed	-
		(*d*) If applicable, explain how loss to follow-up was addressed	-
	Statistical methods	(*e*) Describe any sensitivity analyses	-
	**Results**		
13*		(a) Report numbers of individuals at each stage of study—eg numbers potentially eligible, examined for eligibility, confirmed eligible, included in the study, completing follow-up, and analysed	3-4
		(b) Give reasons for non-participation at each stage	3-4
	Participants	(c) Consider use of a flow diagram	-

14*		(a) Give characteristics of study participants (eg demographic, clinical, social) and information on exposures and potential confounders	3-5
		(b) Indicate number of participants with missing data for each variable of interest	-
	Descriptive data	(c) Summarise follow-up time (eg, average and total amount)	3-7
15*	Outcome data	Report numbers of outcome events or summary measures over time	3-7
16		(*a*) Give unadjusted estimates and, if applicable, confounder-adjusted estimates and their precision (eg, 95% confidence interval). Make clear which confounders were adjusted for and why they were included	3-7
		(*b*) Report category boundaries when continuous variables were categorized	3-7
	Main results	(*c*) If relevant, consider translating estimates of relative risk into absolute risk for a meaningful time period	
17	Other analyses	Report other analyses done—eg analyses of subgroups and interactions, and sensitivity analyses	4-7
	**Discussion**		
18	Key results	Summarise key results with reference to study objectives	7-8
19	Limitations	Discuss limitations of the study, taking into account sources of potential bias or imprecision. Discuss both direction and magnitude of any potential bias	8
20	Interpretation	Give a cautious overall interpretation of results considering objectives, limitations, multiplicity of analyses, results from similar studies, and other relevant evidence	8
21	Generalisability	Discuss the generalisability (external validity) of the study results	8
	**Other information**		
22	Funding	Give the source of funding and the role of the funders for the present study and, if applicable, for the original study on which the present article is based	9

*Give information separately for exposed and unexposed groups.

Table S2. Identification of risk factors for death: univariate analysis (selected parameters; n = 188)

Variable	B-coefficient	P-value	HR	95% Confidence interval	
				Lower	Upper
Procalcitonin on day two	0.008	0.006	1.01[a]	1.00	1.01
Procalcitonin on day three	0.008	0.002	1.01	1.00	1.01
Procalcitonin on day four	0.011	<0.001	1.01	1.01	1.02
Procalcitonin on day five	0.016	0.001	1.02	1.01	1.03
Procalcitonin on day six	0.016	0.091	1.02	1.00	1.04
Procalcitonin on day seven	0.028	0.014	1.03	1.01	1.05
Fibrinogen on day two	- 0.002	0.041	1.00	1.00	1.00
Fibrinogen on day three	- 0.004	0.002	1.00	1.00	1.00
Fibrinogen on day four	- 0.002	0.072	1.00	1.00	1.00
Fibrinogen on day five	- 0.001	0.237	1.00	1.00	1.00
Fibrinogen on day six	- 0.002	0.025	1.00	1.00	1.00
Fibrinogen on day seven	- 0.002	0.051	1.00	1.00	1.00
Resuscitation before ECMO	0.462	0.031	1.59	1.04	2.41
Haemorrhage	0.494	0.021	1.64	1.08	2.50
Arterial thrombosis	0.434	0.078	1.54	0.95	2.50
SAPS III score	0.032	<0.001	1.03	1.02	1.05

[a]For every increase in one unit of measurement, hazard ratio increased by 1%. Abbreviations: ECMO, extracorporeal membrane oxygenation; CI, confidence Intervals; HR, hazard ratio; SAPS III: simplified acute physiology score III

Table S3. Identification of risk factors for death: multivariate analysis (Procalcitonin on day three; n = 188)

Variable	B-coefficient	P-value	HR*	95% Confidence interval	
				Lower	Upper
Procalcitonin	0.007	0.005	1.008	1.002	1.013
Bleeding event during or after ECMO	0.541	0.036	1.718	1.036	2.849
Resuscitation before ECMO initiation	0.113	0.678	1.120	0.656	1.913

*For every increase in one unit of measurement, hazard ratio increased by 1%. Model values for Procalcitonin for day four. Abbreviations: ECMO, extracorporeal membrane oxygenation; CI, confidence Intervals; HR, hazard ratio.

Table S4. Identification of risk factors for death: multivariate analysis (Procalcitonin on day four; n = 188)

Variable	B-coefficient	P-value	HR*	95% Confidence interval	
				Lower	Upper
Procalcitonin	0.009	0.003	1.009	1.003	1.016
Bleeding event during or after ECMO	0.547	0.061	1.729	0.976	3.062
Resuscitation before ECMO initiation	0.189	0.543	1.208	0.657	2.219

*For every increase in one unit of measurement, hazard ratio increased by 1%. Model values for Procalcitonin for day four. Abbreviations: ECMO, extracorporeal membrane oxygenation; CI, confidence Intervals; HR, hazard ratio.

Table S5. Identification of risk factors for death: multivariate analysis (Procalcitonin on day five; n = 188)

Variable	B-coefficient	P-value	HR*	95% Confidence interval	
				Lower	Upper
Procalcitonin	0.013	0.011	1.013	1.003	1.023
Bleeding event during or after ECMO	0.703	0.017	2.021	1.131	3.609
Resuscitation before ECMO initiation	0.273	0.365	1.313	0.728	2.370

*For every increase in one unit of measurement, hazard ratio increased by 1%. Model values for Procalcitonin for day four. Abbreviations: ECMO, extracorporeal membrane oxygenation; CI, confidence Intervals; HR, hazard ratio.
